# Design, Synthesis, Investigation, and Application of a Macromolecule Photoswitch

**DOI:** 10.3389/fchem.2019.00086

**Published:** 2019-02-28

**Authors:** Juan Pang, Ziyu Gao, Huaping Tan, Xincheng Mao, Huiming Wang, Xiaohong Hu

**Affiliations:** ^1^School of Material Engineering, Jinling Institute of Technology, Nanjing, China; ^2^Biomaterials for Organogenesis Laboratory, School of Materials Science and Engineering, Nanjing University of Science and Technology, Nanjing, China

**Keywords:** azobenzene, photoswitch, copolymer, light responsive property, functionalization

## Abstract

Azobenzene (AZO) has attracted increasing interest due to its reversible structural change upon a light stimulus. However, poor fatigue durability and the photobleaching phenomenon restricts its further application. Herein, the AZO domain as a pendent group, was incorporated into copolymers, which was synthesized by radical copolymerization in the research. Structure-properties of synthesized copolymer can be adjusted by monomer ratios. Emphatically, responsive properties of copolymer in different solutions were investigated. In the DMSO solution, copolymer exhibited effective structural change, stable rapid responsive time (1 min) upon UV light at room temperature, stable relative acceptable recovery time (100 min) upon white light at room temperature, and good fatigue resistance property. In an aqueous solution, even more controllable responsive properties and fatigue resistance properties for copolymer were verified by results. More pervasively, the recovery process could be controlled by light density and temperature. In order to clarify reasons for the difference between the AZO molecule and the AZO domain of copolymer, energy barrier or interactions between single atoms or even structural units was calculated using the density functional theory (DFT). Furthermore, the status of copolymer was characterized by dynamic light scattering (DLS) and transmission electron microscope (TEM). Finally, copolymer was further functionalized with bioactive protein (concanavalin, ConA) to reduce the cytotoxicity of the AZO molecule.

## Introduction

Photo molecular switches can reversibly exhibit different properties due to their structural changes upon a light stimulus, making them new favorites in recent decades in fields that require switching effects, such as information science and chemical sensing (Sun et al., 2012; Yuan et al., 2014; Chiang and Chu, 2015; Kim et al., 2015; Bian et al., 2016a). Generally, photo switches possess a conjugated structure and precise spatiotemporal control such as chiral helicene, azobenzene, diarylethene, spiropyran, binaphthyl compounds (Beharry and Woolley, 2011; Sun et al., 2012; Yuan et al., 2014; Chiang and Chu, 2015; Kim et al., 2015; Bian et al., 2016a; Lubbe et al., 2017; Chen et al., 2018; Lerch et al., 2018). Among these molecules, azobenzene's have been intensively investigated on account of their flexible property (Chen et al., 2009, 2018; Schmidt et al., 2010; Beharry and Woolley, 2011; Li et al., 2014; Yuan et al., 2014; Deka et al., 2015; Bian et al., 2016a,b; Lin et al., 2016; Lubbe et al., 2017; Lerch et al., 2018). Upon UV light stimulation, azobenzene can isomerizes from the trans-form to the cis-form (Henzl et al., 2006). Upon visible light or slight heat, cis-form of azobenzene is reversely recovered to trans-form (Henzl et al., 2006). Unfortunately, cis-form of azobenzene is unstable in a natural state (Pang et al., 2018). In actual application, photo switches should satisfy the following: certain thermostability, fatigue durability, detectability, and non-destructive readability. From previous research, azobenzene could not satisfy thermostability and fatigue durability, which restricted its application as a photo switch (Pang et al., 2018). Attempting to solve the problem, many efforts including our research have been applied to design new azobenzenes through all kinds of substitutions at opposite position to improve their properties as photo switches (Pang et al., 2014, 2015, 2018; Ye et al., 2016). Besides these reports, visible light driven azobenzene-based photo-switching molecules have also previously been theoretically designed by different substitute groups (Pang et al., 2014, 2015; Ye et al., 2016).

Although these previous methods successfully solved the problem partly, not all influencing factors had been clarified for the application of azobenzene. For example, polarity of solvents have an influence on the transition of trans-to-cis and the stability of cis-form, due to stacking between molecules, which induces azobenzene to lose its photo switch characteristic (Pang et al., 2018). Moreover, small azobenzene molecules have some cytotoxicity. Such drawbacks restrict its application in many fields, especially biomedicine-related fields. Polymers have less toxicity than small molecules with similar structures. Therefore, azobenzene-based copolymers were designed using an AZO unit as a pendent group, in view of the movability at one end in the research. Radical polymerization is a good way to synthesize copolymer, which has also been used in the research. In order to solve the stacking problem in water, other monomers should be introduced to the azobenzene-based polymer as a chain diluter to enlarge the distance between azobenzene molecules preventing their stacking. In order to broaden its application in the biomedicine-related field, hydrophilicity should be considered. On the one hand, a hydrophilic structure would help copolymer to better disperse in an aqueous solution. On the other hand, hydrophilic biocompatibility is generally good. Therefore, in view this (Hu and Gong, 2016), the hydrophilic monomers HEMA and NVP were chosen to polymerize with the azobenzene monomer.

Furthermore, research also focuses on the functionalization of macromolecules (Tseng et al., 2016; Cao et al., 2018; Wu et al., 2018). Although methods for macromolecule modification are numerous, a flexible, moderate, and a reliable reaction is still an effective and efficient method of polymer modification. Therefore, a functional group was simultaneously introduced into copolymer for further modification during the synthesis of copolymer.

As a whole, the novelty of the research is to provide an available and widely-applied photoswitch copolymer. Therefore, in this study, systematic light response performances and preliminary *in vitro* cytotoxic evaluation for synthesized copolymer, in different environments, were investigated.

## Experiment

### Materials

N-hydroxysuccinimide (NHS), acryloyl chloride, p-aminoazobenzene (AZO), concanavalin (conA), and 2-morpholinoethanesulfonicacid (MES) were purchased from Aladdin. Dichloromethane (DCM), diethyl ether, tetrahydrofuran (THF), dioxane, benzoyl peroxide (BPO), triethylamine (TEA), and dimethyl sulfone (DMSO) were obtained from Sinopharm Chemical Reagent Co., Ltd, China. Trypsin, Dulbecco's modified Eagle's medium (DMEM), fluorescein diacetate (FDA) and 3-(4, 5-dimethyl) thiazol-2,5-dimethyl tetrazolium bromide (MTT) were obtained from Sigma. Fetal bovine serum (FBS) was purchased from Sijiqing biotech. Co., China. All other reagents and solvents were of analytical grade and used as received.

### Synthesis and Characterization of AZO and NAS Monomer

The AZO and NAS monomer was synthesized by acyl chlorination, Briefly, 10 mM AZO or NHS was dissolved in 10 mL anhydrous DCM, into which 11 mmol TEA was added at stirred state. Eleven millimolar acryloyl chloride in 10 mL anhydrous DCM was subsequently dropped into the above solution within 1 h under an ice-water bath. Then the ice-water bath was withdrawn, and the reaction solution was sealed and continued to react for 4–5 h at room temperature. After the reaction was finished, the resultant reaction solution was filtered, washed with saturated NaCl solution and separated by separating funnel for several times to remove salt and unreacted reactants. Finally, the product was obtained by reduced pressure distillation. Final products were characterized by_1_H nuclear magnetic resonance (_1_H NMR, Bruker, AV300) using CDCl_3_ as the solvent.

### Synthesis and Characterization of AZO-HEMA-NVP-NAS Copolymer

Copolymers were synthesized by radical polymerization. Briefly, each monomer with a certain ratio according to Table 1 was added to a round-bottom flask one by one, into which 30 mL dioxane was added to dissolve 10 mmol monomers. After monomers dissolved, 5 mL 0.1 mmol/mL BPO/dioxane solution was added to the reactive solution. Then, nitrogen was inlet into the solution to get rid of oxygen for 15 min before the solution was sealed. The sealed solution was reacted at 70°C for 24 h. The final product was precipitated by diethyl ether and dissolved by THF several times to purify resultant copolymer. Finally, copolymers were obtained by freeze-drying (−50°C 7–8 Pa). Final products were characterized by _1_H nuclear magnetic resonance (_1_H NMR, Bruker, AV300) using DMSO as a solvent and differential scanning calorimetry detection (PerkinElmer, DSC 8500).

**Table 1:** The feed ratio of monomer for copolymers.

	**AZO**	**HEMA**	**NAS**	**NVP**
Copolymer 1 (mol%)	17	42	16	25
Copolymer 2 (mol%)	10	40	10	40
Copolymer 3 (mol%)	5	50	5	40

### Responsive and Recovery Performance of Copolymer

Chosen copolymer was dissolved in DMSO to obtain copolymer dilute solution, which was tracked by UV spectroscopy (Cary 50). A UV lamp (10 W) was used as a photo source to induce trans-to-cis transition of the AZO domain. After UV irradiation, a white light of 685 mW/cm^2^ was used to induce cis-to-trans recovery at room temperature. In order to track the structural change of molecules, real-time UV spectra as a function of irradiation time and recovery time was recorded. Repeated irradiation and recovery methods were applied to demonstrate the fatigue resistance of molecules.

Dilute copolymer aqueous solution was obtained by dilution of copolymer DMSO solution and tracked by UV spectroscopy to investigate response and recovery performance. Besides the above-mentioned performance in the DMSO solution, effects of light density and temperature on cis-to-trans recovery time was also tracked and recorded. Furthermore, the macromolecule status in the water solution was characterized by dynamic light scattering (DLS, nano ZS) and transmission electron microscope (TEM, Philips, Tecnai 12). Transparency of copolymer aqueous solution as a function of pH value was recorded by UV spectroscopy.

### DFT Calculations

All calculations were carried out with (Frisch et al., 2009) Gaussian 09 programs at the CAM-B3LYP/6-31G (d,p) level. The electrostatic potentials of molecules were exhibited, in which positive and negative regions appeared red and blue, respectively (Ransil, 1961). The interaction energy between cis-AZO and HEMA, ΔEcis-AZO···HEMA, was calculated as:Δ*E*_*cis*−*AZO*···*HEMA*_ = *E*_*total*_−(*E*_*cis*−*AZO*_+*E*_*HEMA*_). The basis set superposition error (BSSE) was corrected by counterpoise method (Boys and Bernardi, 1970) in the binding energies calculations.

### Preliminary Evaluation of Copolymer for Biomedical Application

ConA was used to functionalize the chosen copolymer in a simple method. Briefly, ConA was added into 0.1% copolymer aqueous solution with a concentration of 5 mg/mL under stirred state and reacted with NAS domain for 24 h. the resultant product was purified using the above-mentioned method (diethyl ether/THF) and obtained by freeze-drying for further use.

HUVEC cells were incubated in a humidified atmosphere of 95% air and 5% CO_2_ at 37°C. The used cells were detached using 0.25% trypsin in PBS for the experiment. Simultaneously, AZO monomer, copolymer and copolymer-ConA were dispersed in DMEM with certain concentrations separately with the same molar ratio (AZO domain). Then 100 μL of the above-mentioned solution were added into each well of the 96-well culture plate, into which the 100 μL cell suspension containing 20,000 cells were subsequently added. Cytotoxicity was evaluated by MTT assay after cells were cultured for 24 h. Briefly, after 20 μL MTT was incubated with cells for 4 h, 200 μL DMSO was added to dissolve the formed formazan pigment. The absorbance of 150 μL above solution at 560 nm was recorded by a microplate reader (Infinite M200 PRO) (Pang et al., 2018).

### Statistical Analysis

Data were analyzed using the *t*-test for differences. Results were reported as means ± standard deviation. The significant level was set at *p* < 0.05.

## Results and Discussion

### Synthesis and Characterization of AZO Monomer and NAS Monomer

**Figure 1:**
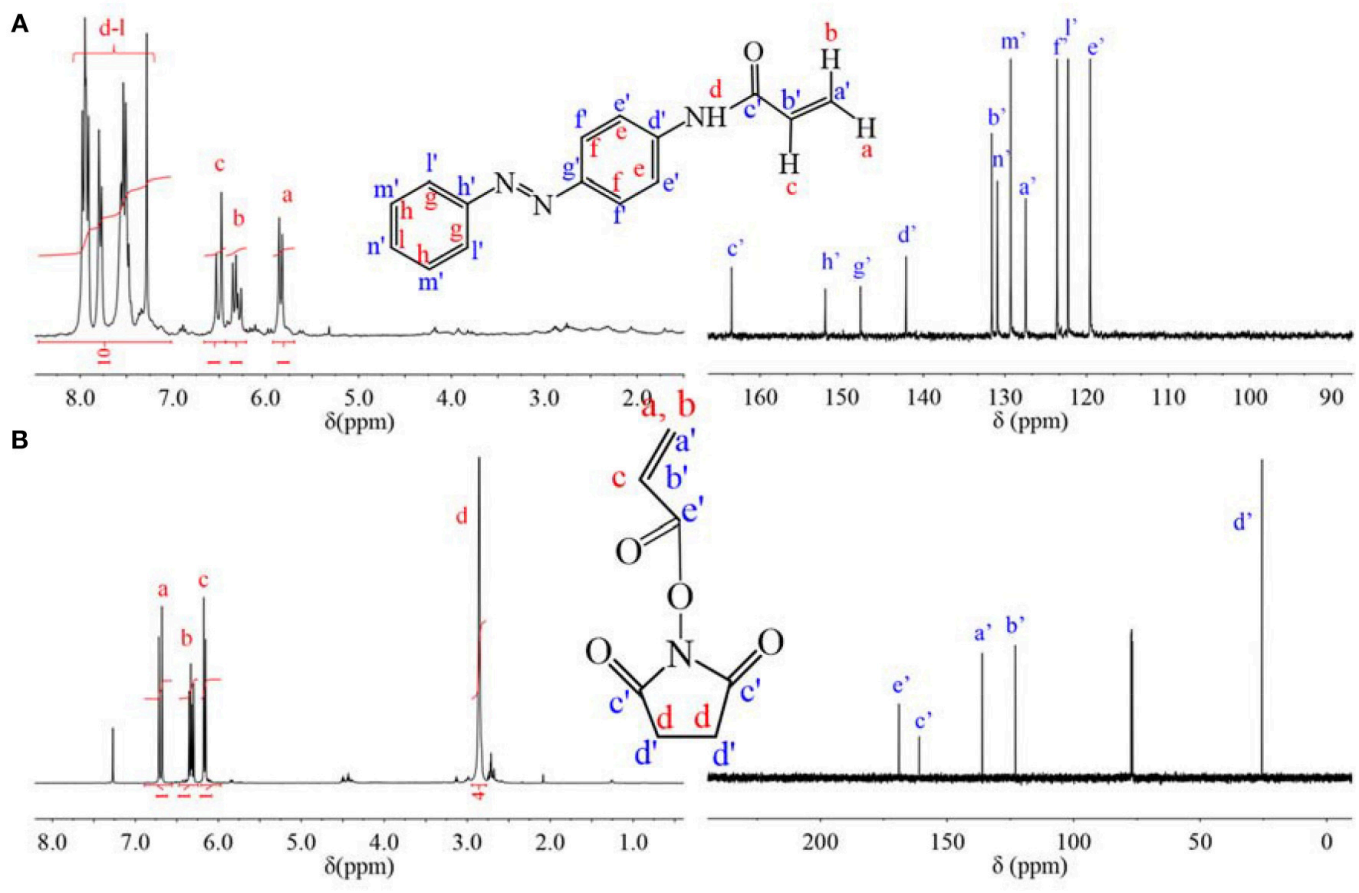
**(A)**_1_H NMR spectrum and _13_C NMR spectrum of AZO monomer. **(B)**_1_H NMR spectrum and _13_C NMR spectrum of NAS monomer.

C=C group was modified onto AZO and NHS molecules, respectively through acyl chlorination. The chemical information of monomer was confirmed by _1_H NMR spectrum and _13_C NMR spectrum in Figure 1. From _1_H NMR spectrum of Figure 1A, chemical shifts from 5.7 to 6.6 ppm were attributed to three H on vinyl group at a, b, c positions, which indicated that vinyl was successfully modified onto AZO molecules. Simultaneously, chemical shifts from 7.1 to 8.2 ppm are attributed to H of the benzene ring and imine groups. According to integration of peak from 7.1 to 8.2 ppm, the number of H on the benzene ring and imine groups was 10, which was consistent with the structure of the AZO monomer in Figure 1A and indicated that the product was pure without any impurities. Besides _1_H NMR spectrum, chemical shifts were attributed to the _13_C NMR spectrum one by one as shown in Figure 1A, which was also consistent with the structure of the AZO monomer. Therefore, NMR spectra verified that the AZO monomer was successfully synthesized.

Similarly, chemical shifts of three H on the vinyl group at a, b, c positions confirmed the successful modification of vinyl on NHS molecule in _1_H NMR spectrum of Figure 1B, chemical shift at 2.9 ppm are attributed to H of the five-membered ring at the d position, which was integrated to 4, that was, the number of H on five-membered ring was 4. Additionally, chemical shifts of _13_C NMR spectrum were attributed one by one as shown in Figure 1B, which was also consistent with the structure of NAS. Thus, successful synthesized NAS was proven by NMR spectra.

### Synthesis and Characterization of AZO-HEMA-NVP-NAS Copolymer

**Figure 2:**
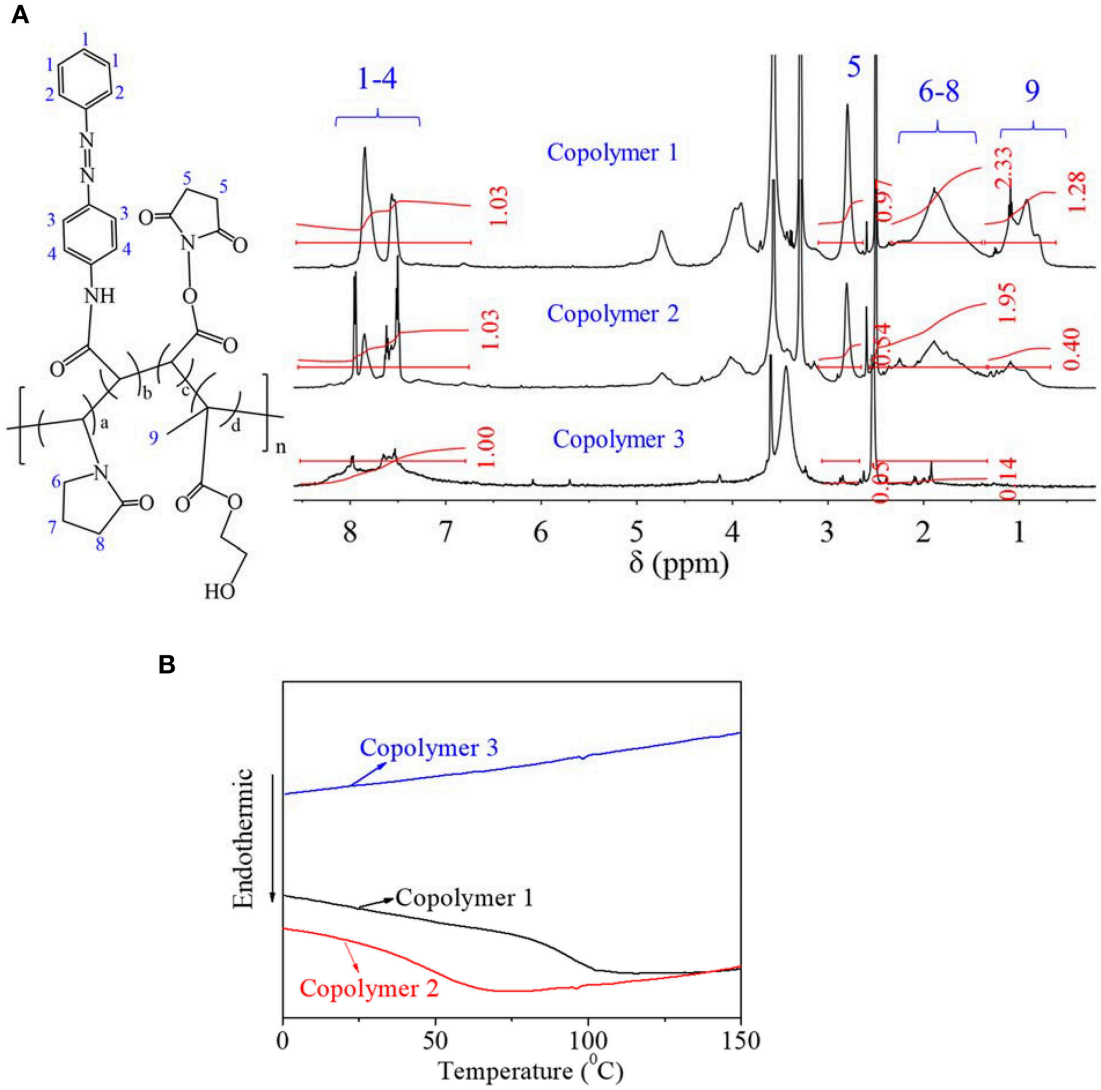
**(A)**_1_H NMR spectra of copolymers. **(B)** DSC curve of copolymers.

Synthesized AZO-HEMA-NVP-NAS copolymers were characterized by _1_H NMR spectrum in Figure 2A. The details of chemical shift are listed as follows: chemical shifts from 7.4 to 8.5 ppm are attributed to the protons of the benzene ring on the AZO domain at 1–4 position, chemical shift at 2.7 ppm is attributed to the protons of the five-membered ring on the NAS domain at 5 position, chemical shifts from 1.2 to 2.3 ppm are attributed to the protons of the five-membered ring on NVP domain at 6–8 position, chemical shifts from 0.5 to 1.2 ppm are attributed to the protons of methyl on the HEMA domain at 9 position. Therefore, four different units of copolymer was confirmed by NMR spectra. Besides qualitative analysis, _1_H NMR spectrum provided quantitative information since areas of resonance peaks are proportional to the number of protons. According to areas in Figure 2A at different positions, relative domain content could be calculated by the average proton intensity ratio, which is listed in Table 2. Compared with the feed ratio of monomers, less AZO monomer entered the polymer chain for copolymer 1. Adversely, when the AZO feeding ratio was increased, more AZO monomer entered the polymer chain. Simultaneously, copolymer yield of copolymer 2 and copolymer 3 was much less than that of copolymer 1, from the experimental results. It was inferred that three other monomers, especially HEMA, were not easily copolymerized with the AZO monomer when the AZO feed ratio was decreased to 10 and 5. Moreover, when the AZO feed ratio was larger than 20, more unpolymerized AZO monomer could be detected in the polymerized system. In addition, copolymer 1 could dissolve in DMSO and has a certain solubility in water when using DMSO as a cosolvent, but copolymer 2 and copolymer 3 only slightly dissolved in DMSO and could not dissolve in water.

**Table 2:** Structural information of copolymers.

	**Structural unit proportion**				**M_**P**_**
	**AZO**	**HEMA**	**NAS**	**NVP**	
Copolymer 1 (mol%)	9	37	21	33	2,455
Copolymer 2 (mol%)	16	19	19	46	1,588
Copolymer 3 (mol%)	75	0	9	16	1,299

In order to clarify the structural characteristics of copolymers, the DSC curve for copolymers was detected and shown in Figure 2B. For copolymer 1, obvious T_g_ transition was found around 80–90°C; for copolymer 2, T_g_ transition was reduced to 50°C; for copolymer 3, T_g_ transition was dismissed, which indicated that the AZO domain on the polymer chain had no obvious T_g_ transition. From our previous research, T_g_ of pHEMA was around 110°C and T_g_ of PVP was around 130°C (Roorda et al., 1988; Xiang and Anderson, 2005). Therefore, it was inferred that the length of either the HEMA domain or the NVP domain on the copolymer chain was shorter than the normal length of their homopolymers, since the T_g_ transition was directly dependent on the domain length of the polymers. Based on the above-mentioned discussion and analysis, copolymer 1 was chosen for further investigation.

### Responsive Performance and Characterization of Copolymer

**Figure 3:**
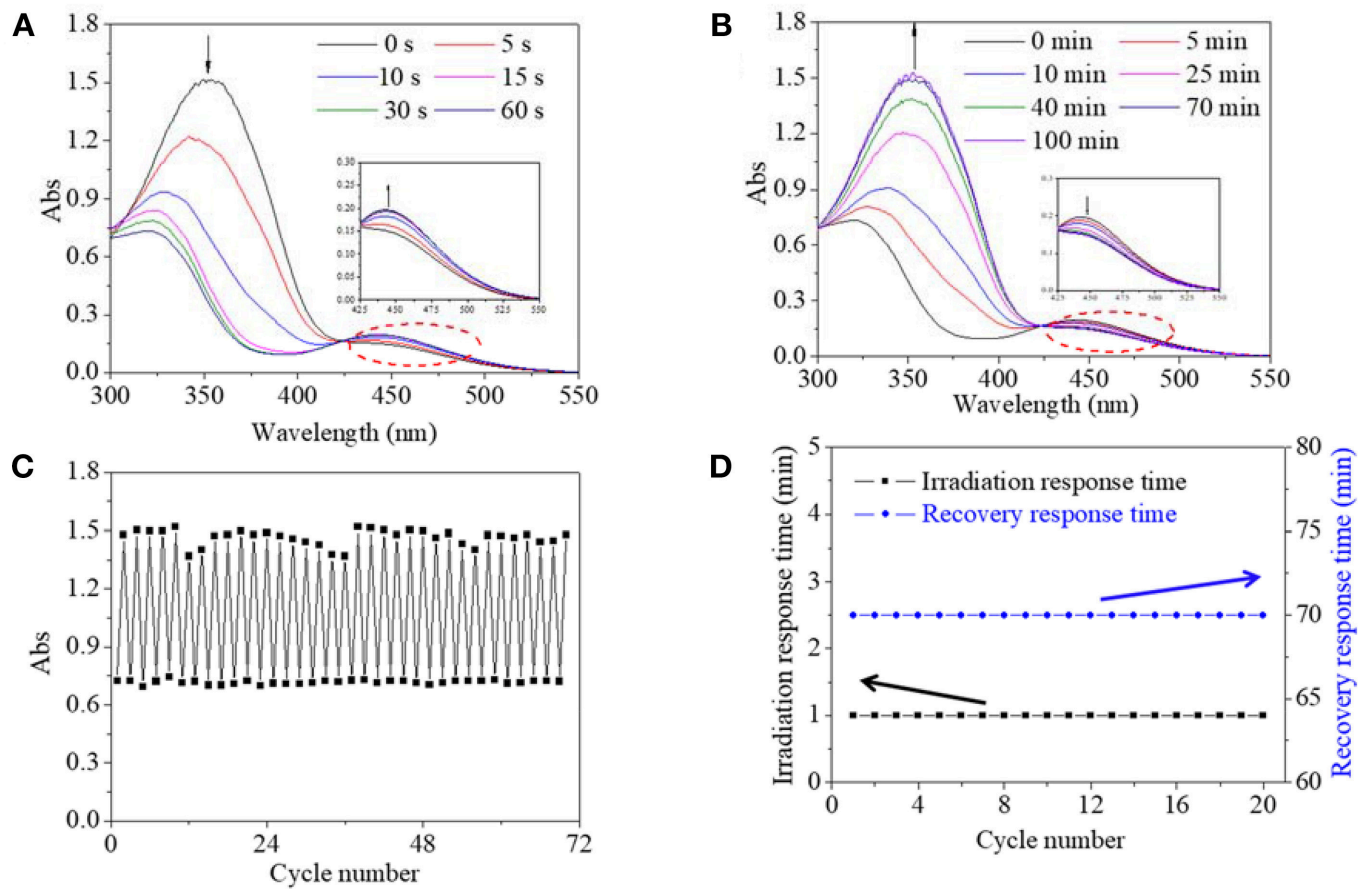
UV spectra of copolymer 1 DMSO solution as a function of irradiation time **(A)** and recovery time **(B)**. **(C)** Absorbance at 360 nm/346 nm of copolymer 1 DMSO solution as a function of cycle number. **(D)** Irradiation response time under UV irradiation and recovery response time under 685 mW/cm^2^ white light and room temperature as a function of cycle number.

Since the AZO molecule responded to UV light, UV light was used to induce the isomerization transition of the AZO molecule or domain, which was reflected in the UV spectrum of their dilute solution from the verified theory and previous research. Firstly, UV spectra of the copolymer DMSO dilute solution, as a function of irradiation time and recovery time, were tracked in Figures 3A,B. Before UV irradiation, a maximum absorption peak at 360 nm belonging to the π-π^*^ transition and a small flat absorption peak at 450 nm belonging to the n-π^*^ transition was observed (Figure 3A), which were, respectively attributed to trans-isomer and cis-isomer of the AZO domain on the polymer chain. Upon UV irradiation, the maximum absorbance at 360 nm decreased significantly and shifted to 346 nm, and the absorbance at 450 nm increased slightly with irradiation time until 60 s, which indicated that the trans-form had been transferred to the cis-form (Figure 3A). The slight blue shifted from 360 to 346 nm which might be attributed to the copolymer structural response upon UV light stimulations, since the phenomenon was not found in AZO small molecules according to our previous research (Pang et al., 2018). Upon white light irradiation, absorbances at 346 nm were gradually shifted to 360 nm and recovered to their respective origin value within 70 min and absorbances at 450 nm were also recovered to their respective origin value, which confirmed the reversible and effective recovery of the trans structure (Figure 3B). Rapid transfer from trans to cis upon photo ensured a quick copolymer response time, and simultaneously the gradual recovered process permitted enough operation time. Secondly, fatigue resistance of copolymer was evaluated by repeated UV/white light irradiation. The absorbance at 360/346 nm of the copolymer DMSO solution as a function of the cycle number is recorded in Figure 3C. It was found that maximum absorbance at 360 nm, either initially or after recovery, stabilized at 1.4–1.5 regardless of the circle time, and simultaneously the minimum absorbance at 346 nm after UV irradiation was stabilized around 0.7 regardless of the circle time (Figure 3C). Moreover, irradiation response time was stable at 1 min and recovery response time was stable at 70 min regardless of the circle time (Figure 3D). These results confirmed that cis-form could be exist with stability after irradiation and the trans-form could be recovered after white light irradiation without any sign of fatigue or photobleaching. Generally, effective structural change, stable rapid responsive time, stable enough/controllable operation time and fatigue resistance are all desirable properties for a photo switch. Therefore, these results revealed that the synthesized copolymer in the DMSO solution exhibited typical characteristics of photo switch, which was superior to an unmodified AZO small molecule with an obvious photobleaching phenomenon and an uncontrollable response/recovery process according to our previous research (Pang et al., 2018).

**Figure 4:**
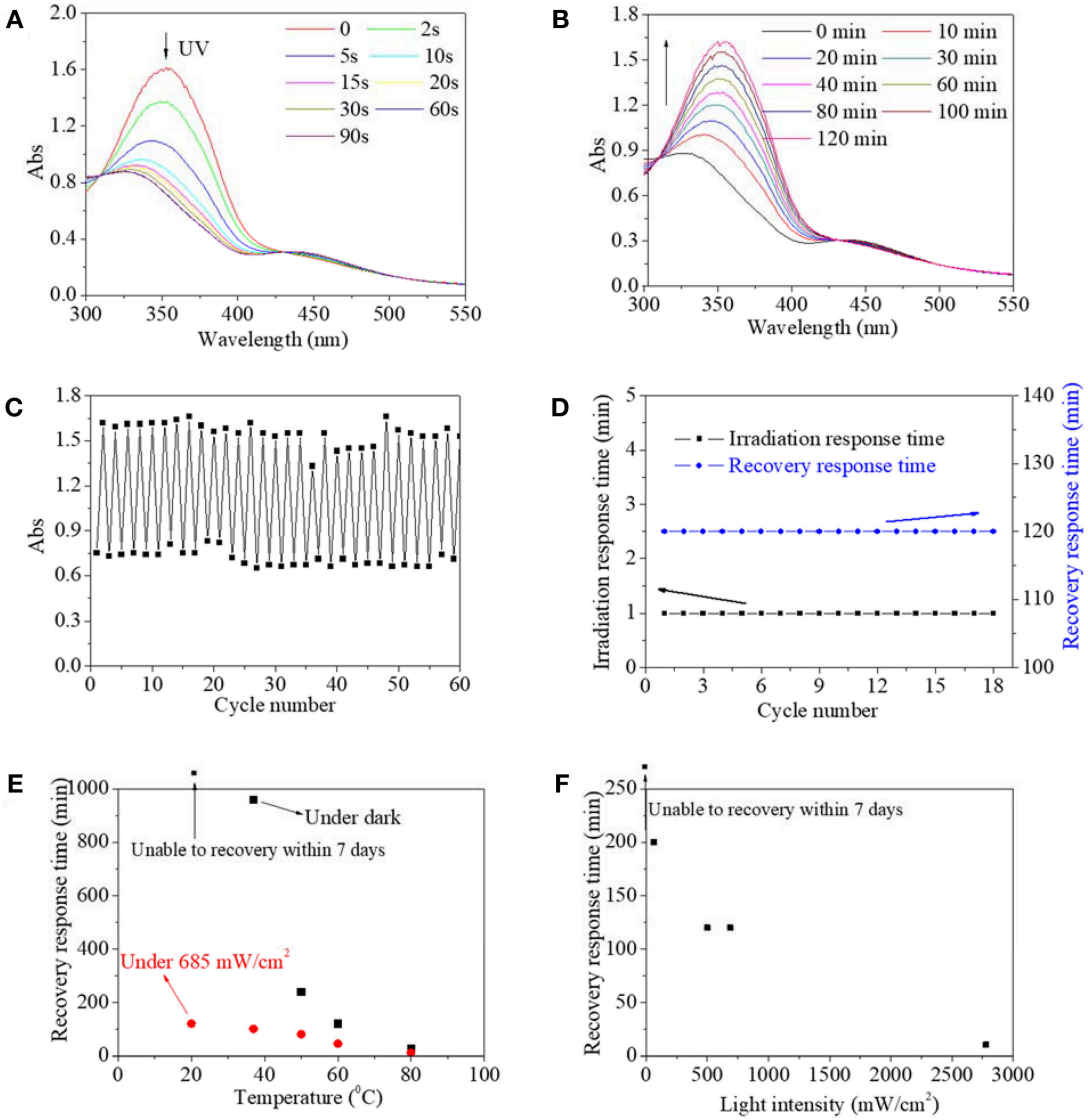
UV spectra of copolymer 1 aqueous solution as a function of irradiation time **(A)** and recovery time **(B)**. **(C)** Absorbance at 360 nm/346 nm of copolymer 1 aqueous solution as a function of cycle number. **(D)** Irradiation response time under UV irradiation and recovery response time under 685 mW/cm^2^ white light as a function of cycle number. The recovery response time as a function of temperature **(E)** and light intensity **(F)**.

Besides an organic solvent, an aqueous solution environment was a more frequently used medium for the actual application of the photo switch. Thus, the characteristics of copolymer in a dilute aqueous solution were also intensively investigated (Figure 4). Not surprisingly, the copolymer aqueous solution possessed the same UV spectra as the copolymer DMSO solution. Similarly, the maximum absorbance at 360 nm decreased significantly and the absorbance at 450 nm increased slightly with irradiation time up to 60 s (Figure 4A), and then absorbances at 360 and 450 nm could be reversibly recovered to their respective origin value within 120 min (Figure 4B). Furthermore, maximum absorbance at 360 nm either at initial or after recovery was stabilized at 1.5–1.7 regardless of the cycle number, and simultaneously, minimum absorbance at 346 nm after UV irradiation was stabilized at 0.7–0.8 regardless of the circle time (Figure 4C). Likewise, irradiation response time was stable at 1 min and recovery response time was stable at 120 min regardless of the circle time (Figure 4D). These results revealed that the synthesized copolymer in an aqueous solution also exhibited typical characteristics of photo switch, which broadened its potential application, especially in biomedical-related fields. As a contrast, on account of their dispersity, unmodified AZO small molecule in water could not respond to UV in a detectable manner, according to our previous research. Since controllable recovery is an ideal state for the application of photo switch, factors including temperature, and light density, to influence the recovery from cis-form to trans-form, were studied (Figures 4E,F). The recovery response time shortened with the increase of temperature regardless of white light irradiation, as shown in Figure 4E. In dark, the recovery from cis-form to trans-form could not be realized after 7 days at 20°C. But when the temperature was increased to 50°C, the recovery response time shortened to a large extent to 5 h. Upon white light irradiation, the recovery response time declined linearly with the increase of temperature. Moreover, the recovery response time under white light was shorter than that in the dark until the temperature reached 80°C. With respect to the effect of white light intensity, the recovery response time significantly shortened with increased light intensity, as shown in Figure 4F. The detailed recovery process is provided in Figures S1–S3.

Theoretically, two ground state for trans- and cis structure, and one excited state were involved in the AZO molecules or domains. A transition from trans to cis must overcome an energy barrier from the trans structure ground state to the excited state, while transition either from cis to trans must overcome an energy barrier from cis structure ground state to excited state (Kathan and Hecht, 2017). It was inferred that UV light could provide enough energy to induce the AZO molecules or domain from the trans structure ground state to the excited state, and similarly heat/white light could provide needed energy to induce the molecule or domain from the cis structure ground state to the excited state. Therefore, it was easy to understand that higher temperature and light intensity resulted in a shorter recovery response time. Generally, the energy of trans form is lower than the energy of cis form, and the trans structure is therefore more stable than the cis structure.

**Figure 5:**
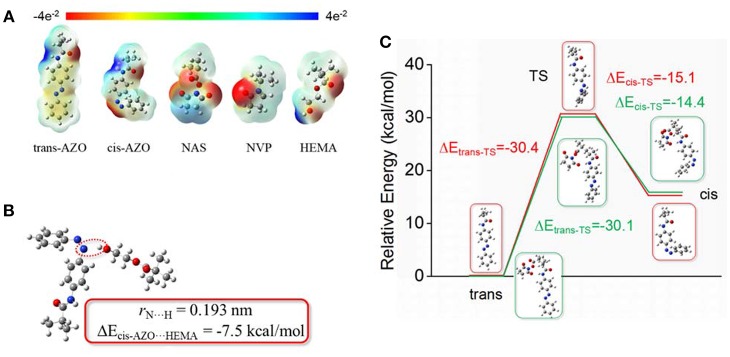
Optimized (calculated) **(A)** Electrostatic potential of all domains. **(B)** Interaction energy between cis-AZO domain and HEMA domain. **(C)** Transition barriers by DFT calculations.

From the above-mentioned results and discussions, the AZO domain in a macromolecule environment, possessed much better stable and controllable recovery property and fatigue durability than the AZO monomer or AZO small molecule, although they had a similar rapid light response property. In order to clarify the microstructural relationship between domains, electrostatic potential, interaction energy, and transition barriers were calculated using DFT calculations, as shown in Figure 5. Through optimization, it was found that the trans-AZO domain exhibited electroneutrality, while the cis-AZO domain exhibited slight electron donor characteristics. Simultaneously, the other three domains exhibited electron donor characteristics due to the existence of O atoms. However, the end of the HEMA domain exhibited some electron acceptor characteristics (Figure 5A). Furthermore, the interaction energy between the cis-AZO domain and the HEMA domain was calculated as −7.5 kcal/mol (Figure 5B), which was not obvious and would not bring a significant interaction between them. In order to verify the inference, two transition barriers between trans base state and excited state (Δ*E*_*trans*−*TS*_) and between excited state and cis base state (Δ*E*_*cis*−*TS*_) were calculated (Figure 5C). It was found that Δ*E*_*trans*−*TS*_ of the AZO domain on the macromolecule environment was nearly the same that that of the AZO monomer, which was also a reason of similar rapid response property upon UV light stimulation. Although Δ*E*_*cis*−*TS*_ of the AZO domain on the macromolecule environment was slightly higher than that of the AZO monomer, the difference between them was not large enough to bring significant performance change. Even from the optimal or calculated results, the cis-form of the AZO domain on the copolymer should be slightly less stable than the AZO monomer, which is in contrast to our results. Therefore, it was inferred that the stable and controllable recovery property of the AZO domain on copolymer, came from some spatial effects due to chain entanglement and even formed cluster formation in the solution, especially in an aqueous solution. In cis spatial construction, a macromolecule cluster was metastable under the combined action of all kinds of interactions including the hydrophobic effect, electrostatic effects and the chain entanglement. Although Δ*E*_*cis*−*TS*_ for the AZO domain itself was not large, recovery transition of cis-to-trans needed to change whole cluster construction, which is a complex system and cannot be calculated using the existent and simple model. Therefore, the realization of this transition needed extra energy besides Δ*E*_*cis*−*TS*_ for the AZO domain.

**Figure 6:**
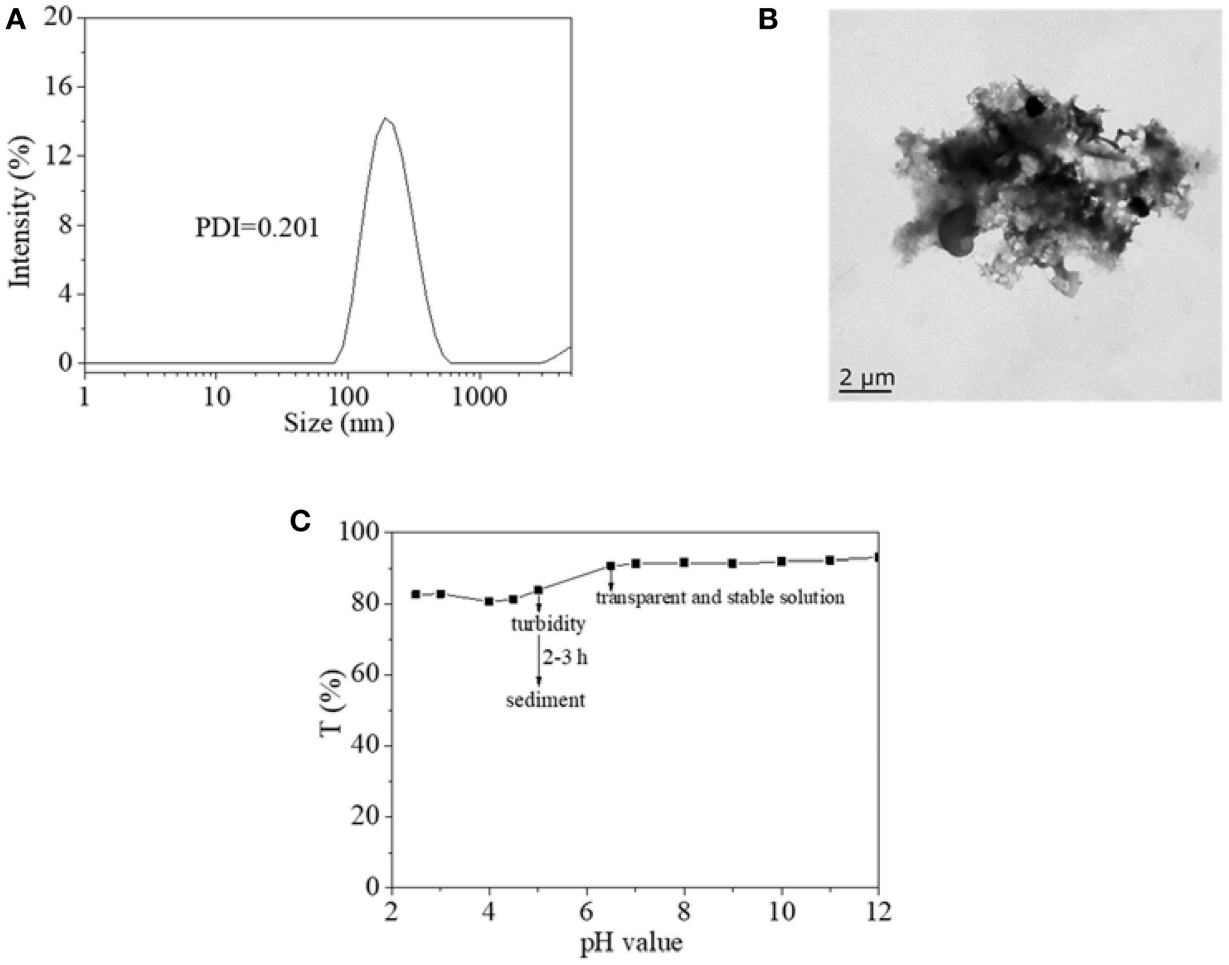
**(A)** Hydrodynamic diameter of copolymer 1 aggregate in water by DLS. **(B)** TEM images of copolymer 1 aggregate in water. **(C)** Transparency of copolymer 1 aqueous solution as a function of pH value.

From the above discussion, the copolymer was an idea material for photoswitch application, especially in an aqueous environment. In order to clarify the properties for further application of copolymer in an aqueous environment, DLS, and TEM were used to clarify its microscopic state in water, as shown in Figure 6. It was found that copolymer presented a kind of nanocluster in water with an effective diameter of around 200 nm (Figure 6A). However, the nanocluster did not form a stable and single shape like nanosphere, nanosheet, even nano fiber when they were dried on copper mesh (Figure 6B). Therefore, it was inferred that nanoclusters in water were unstable and might form due to a weak interaction. Furthermore, the effects of pH value on the aggregation state of copolymer was studied using transparent copolymer solution, which is shown in Figure 6C. The copolymer solution, in neutral or alkaline state, exhibited transparent, and stable homogenous characteristics. Copolymer solution in a mild acid state became turbid after 2–3 h with an obvious sediment of the copolymer. The characteristic endowed copolymer with another pH sensitive property. These results were also verified in the above-mentioned inference of the stable and controllable recovery property of the AZO domain on copolymer.

### Preliminary Evaluation of Copolymer for Biomedical Application

**Figure 7:**
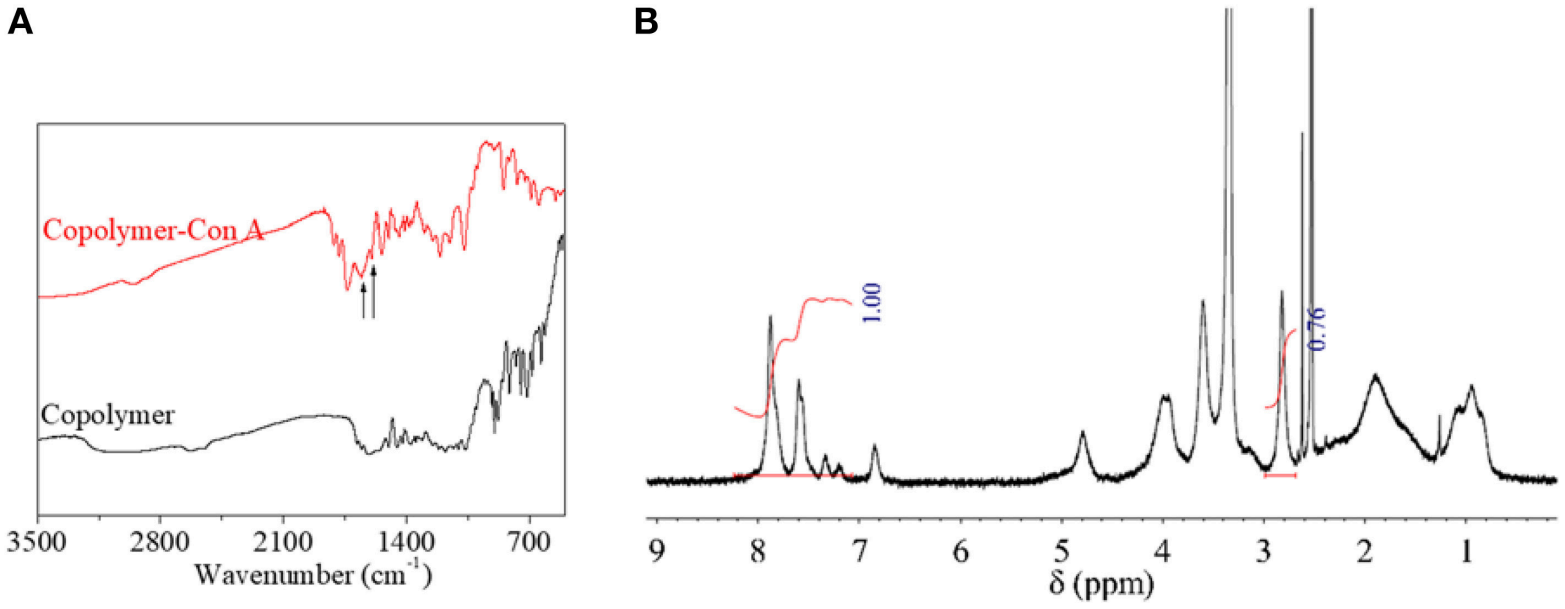
**(A)** IR spectra of copolymer and copolymer-Con A. **(B)**_1_H NMR spectrum of copolymer-Con A.

In order to evaluate the functionalization and biomedical application of copolymer, copolymer was functionalized with bioactive protein (ConA) through a simple reaction between the NAS domain and the amino group of bioactive protein. After functionalization, copolymer-ConA was characterized by IR spectra and _1_H NMR spectrum (Figure 7). On account of similar chemical structures between copolymer and protein, their IR spectra were similar too. But in _1_H NMR spectrum of copolymer-ConA, relative integration of a chemical shift at 2.7 ppm, belonging to the characteristic group on the NAS domain at 5 position in Figure 2 was reduced to 70% of the original value, compared to the AZO domain. This variation verified the successful modification of ConA onto copolymer.

**Figure 8:**
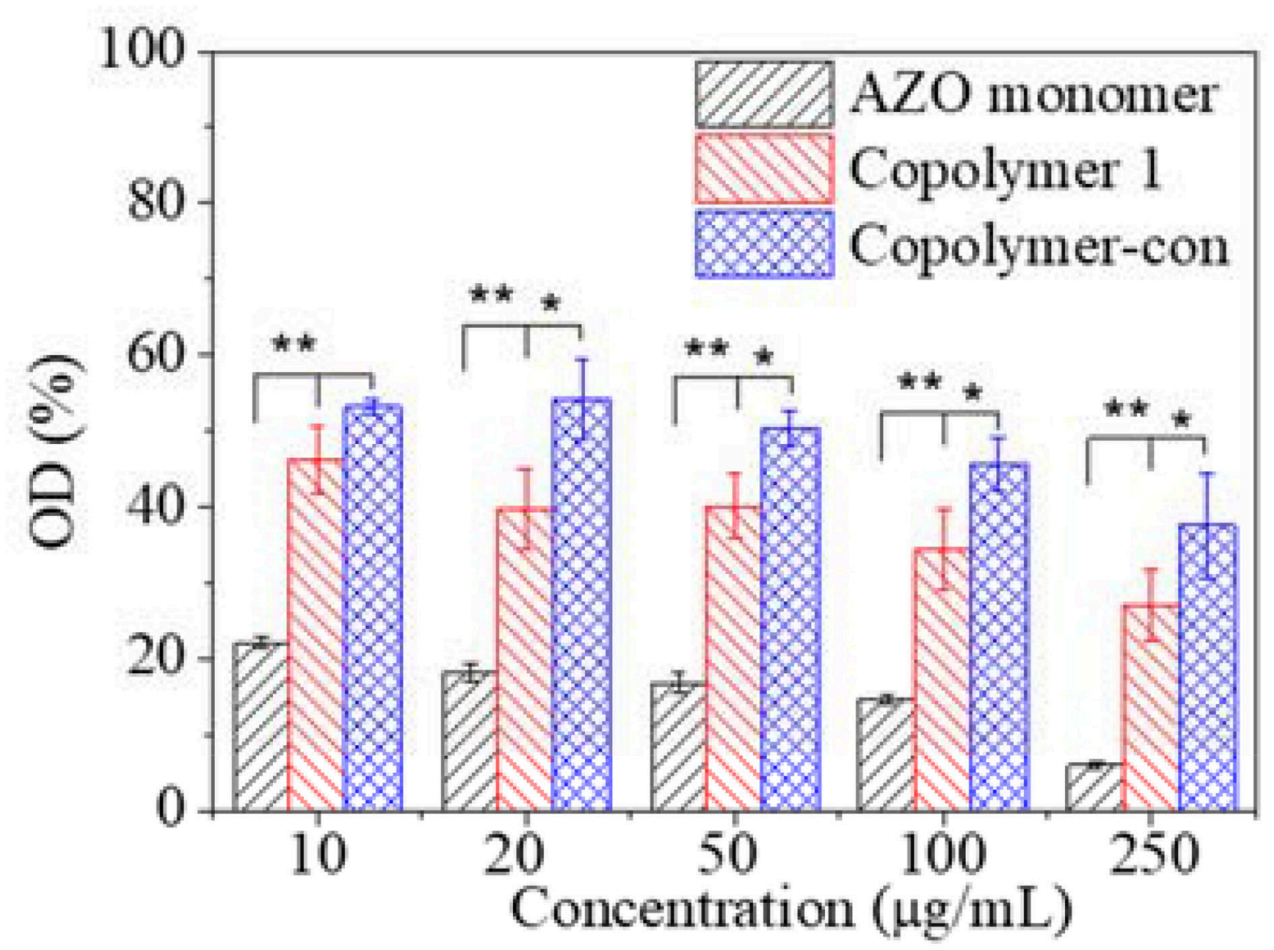
Relative optical density of cells as a function of compound concentration after cultured 24 h and incubated with MTT using TCPs as a control. ^*^p < 0.05; ^**^p < 0.01.

Since cytotoxicity was a primary request for biomedical application, *in vitro* cytotoxicity of copolymer and copolymer-ConA were evaluated using the AZO monomer as a contrast, as shown in Figure 8. The OD of cells decreased with copolymer and copolymer-ConA concentration when their concentration was larger than 100 μg/mL. Furthermore, OD of cells with copolymer-ConA was significantly higher than that with copolymer, regardless of their concentration, which indicated that copolymer-ConA had less cytotoxicity. As a contrast, equivalent AZO exhibited much greater acute cytotoxicity than copolymer and copolymer-ConA with great significance. Although only 50–60% of cells could survive with an even addition of copolymer-ConA, copolymerization of the AZO monomer and functionalization of bioactive protein reduced cytotoxicity of the AZO molecule to large extent. These results revealed that the copolymer could be a suitable material for photoswitch application in the biomedical field.

## Conclusion

AZO monomer and NAS monomer were successfully synthesized by acyl chlorination. AZO-HEMA-NVP-NAS copolymers were successfully synthesized by radical copolymerization with controllable domain content by monomer ratios. Molecular weight and glass transition of copolymer were influenced by monomer ratios. In the DMSO solution, copolymer exhibited effective structural change, stable rapid responsive time (1 min) upon UV light at room temperature and stable relative acceptable recovery time (100 min) upon white light at room temperature. Besides these, fatigue resistance was also observed, which further confirmed the excellent performance of copolymer as a photo switch. In aqueous solutions, even more controllable responsive property and fatigue resistance of copolymer was verified by results. More pervasively, the recovery process could be controlled by the light density and temperature. Results of molecular simulation confirmed that the outstanding performance of copolymer as a photo switch did not come from the change in the energy barrier or from interactions between single atom or even structural units, especially in an aqueous solution. Therefore, it was inferred that controllable recovery property resulted in chain entanglement or even cluster formation. Further DLS results were also confirmed. Moreover, copolymer in an aqueous solution exhibited a pH-dependent property. Finally, copolymer was successfully functionalized with bioactive protein through the simple reaction of preliminary investigation for its application in the biomedical field. Functionalized copolymer possesses lower cytotoxicity, which confirms its application prospect as a photo switch in bio-related fields. Overall, the synthesized copolymer possesses essential requirements of photoswitch and even a prominent performance in an aqueous environment, which affords it good prospects in many fields including the biomedical field.

## Author Contributions

JP designed macromolecule and finished the calculation part. ZG synthesized and characterized copolymers. HT gave helpful suggestion in the part of copolymer functionalization and *in vitro* evaluation. XM finished part of synthesis of monomer and copolymer. HW finished part of copolymer characterization. XH gave the ideas and designed the whole research.

### Conflict of Interest Statement

The authors declare that the research was conducted in the absence of any commercial or financial relationships that could be construed as a potential conflict of interest.
