# Supplementary material for: Design, Synthesis, Investigation, and Application of a Macromolecule Photoswitch
Source: Front Chem. 2019 Feb 28;7:86. doi: 10.3389/fchem.2019.00086 (PMC6403147; doi:10.3389/fchem.2019.00086)
Supplement: Supplementary file 1 [file Data_Sheet_1.PDF]

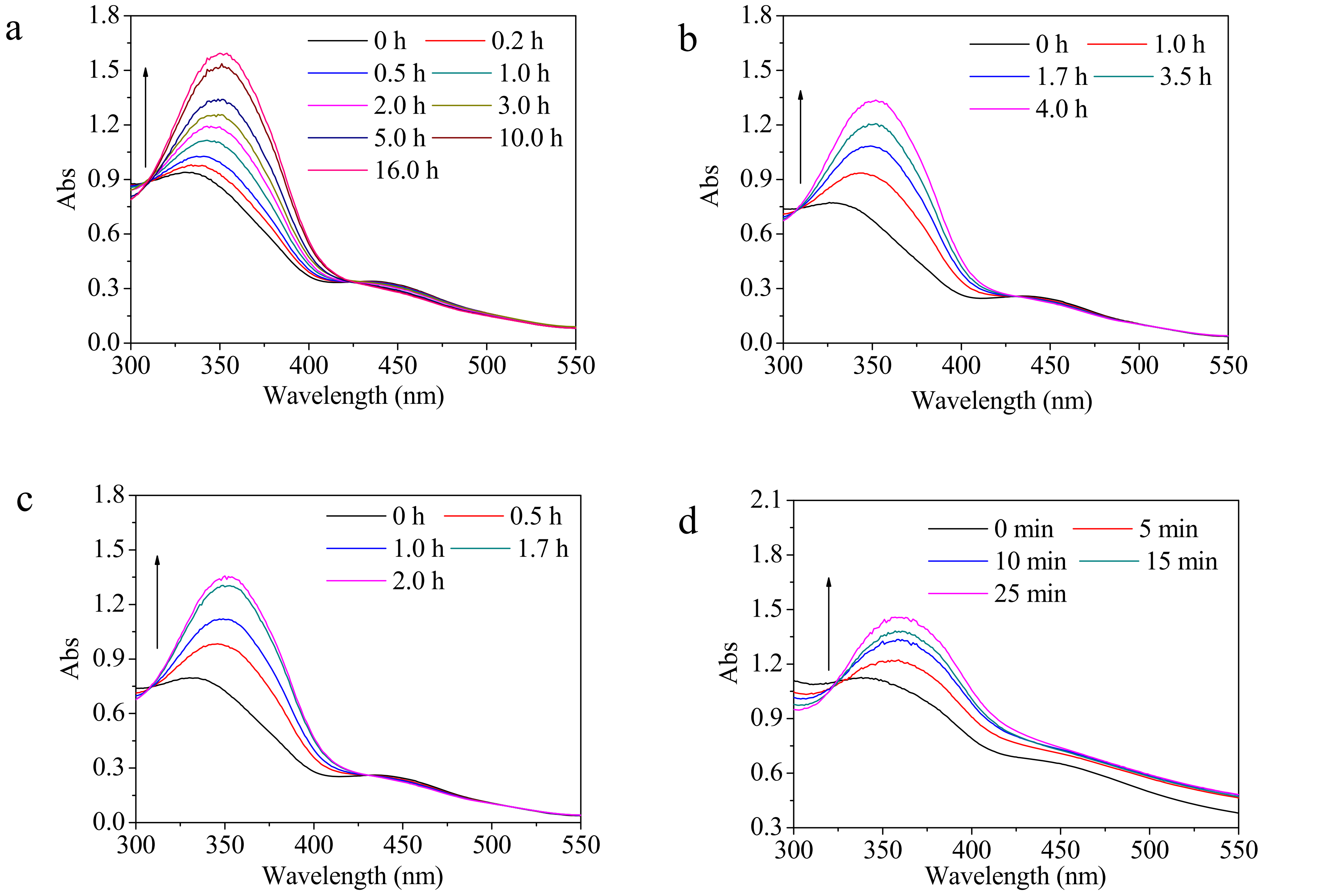

Fig. 1s the recovery process of UV-irradiated copolymer aqueous solution under dark and (a)37^{0}C; (b) 50 ^{0}C; (c) 60 ^{0}C; (b) 80 ^{0}C

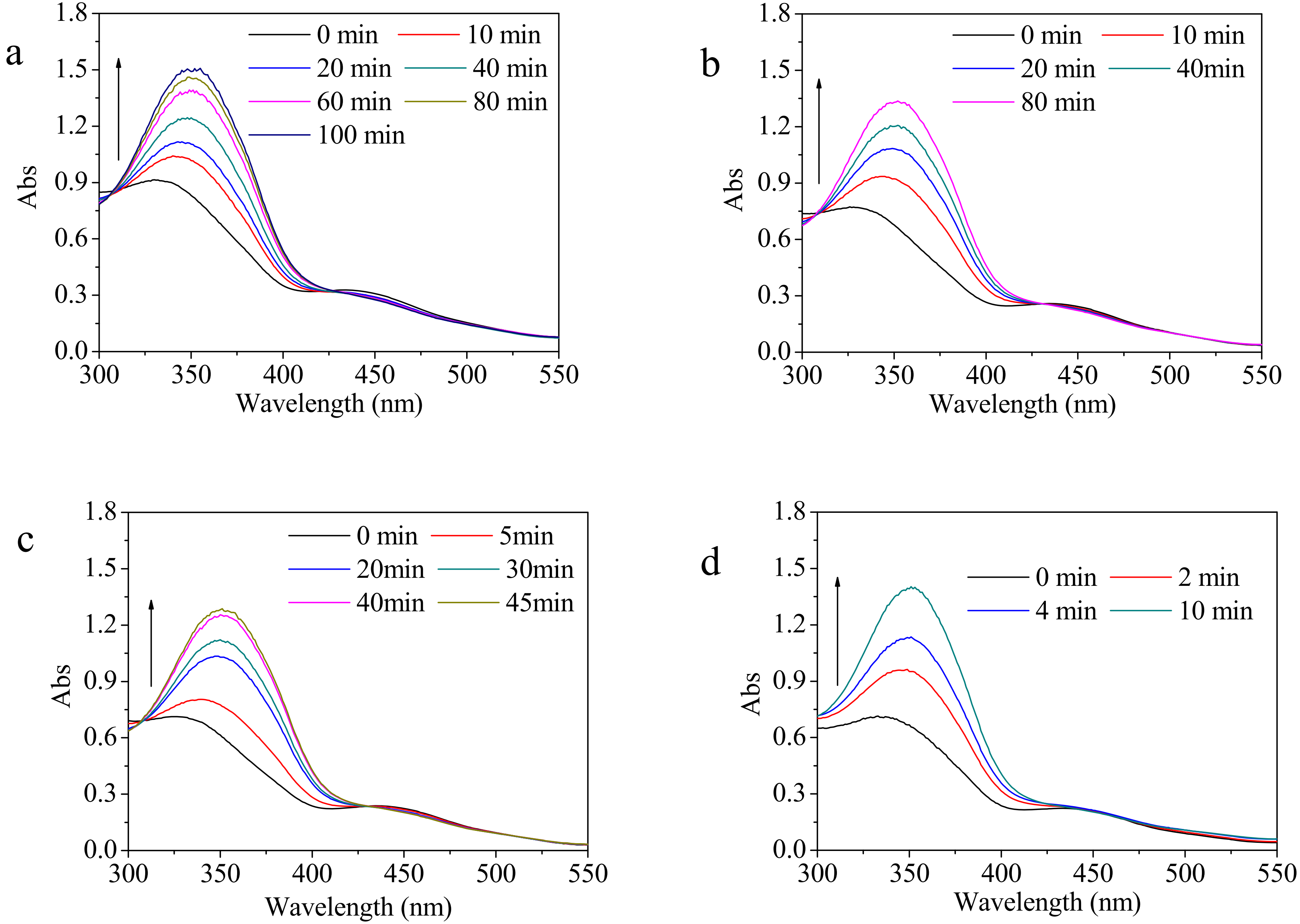

Fig. 2s the recovery process of UV-irradiated copolymer aqueous solution under 685 mW/cm^2 white light and (a)37^0C; (b) 50 ^0C; (c) 60 ^0C; (b) 80 ^0C

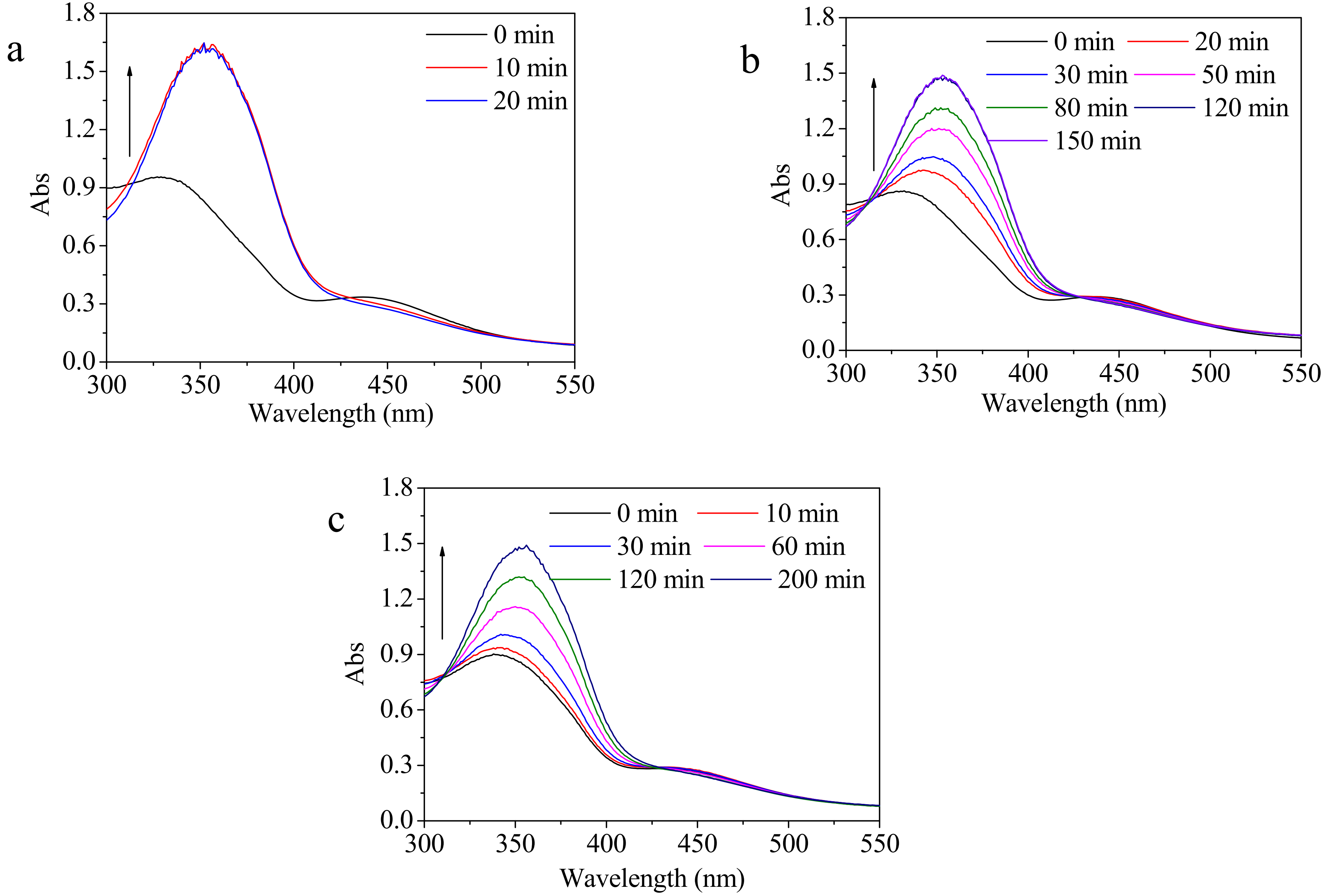

Fig. 3s the recovery process of UV-irradiated copolymer aqueous solution under $20^{0}C$ and (a) 2780 mW/cm^2 ; (b) 500 ^{0}C; (c) 65 ^{0}C.
